# Parke, Davis & Co.

**Published:** 1882-02-20

**Authors:** 


PARKE, DAVIS & CO.—See the new advertisement of this large and excellent house in this issue of the Journal. No end to the push and energy of this establishment.

